# Radiographic Quality of Single *vs.* Multiple-Visit Root Canal Treatment Performed by Dental Students: A Case Control Study

**DOI:** 10.22037/iej.v13i2.19427

**Published:** 2018

**Authors:** Kholod K Al-Manei

**Affiliations:** a *Department of Restorative Dental Sciences, Division of Endodontics, College of Dentistry, King Saud University, Riyadh, Kingdom of Saudi Arabia*

**Keywords:** Case Control, Endodontic Treatment, Multiple Visits, Radiographic Evaluation, Single Visit

## Abstract

**Introduction:**

The aim of this study was to compare the quality of root canal treatment provided by undergraduate dental students in relation to the number of dental visits.

**Methods and Materials:**

Root canal treatments done by 77 dental students were observed. For each student, one tooth treated in a single visit was matched and compared with a tooth treated in multiple visits. The effect of preoperative conditions on the quality of root canal treatment and the number of visits were analyzed. The quality of root canal treatment was determined by the following criteria: obturation length, density, taper, and presence of procedural errors. The data were statistically analyzed using an exact conditional logistic regression test, and the level of significance was set at 0.05.

**Results:**

There was no statistically significant association between single- and multiple-visit root canal treatment in terms of obturation length (*P*=0.263), obturation density (*P*=0.625), and obturation taper (*P*=1.00). The incidence of procedural errors in teeth which required a single visit (7.8%) was less but not significantly different from those treated in multiple visits (16.9%). The presence of preoperative conditions was not significantly associated with multiple-visit treatment.

**Conclusion:**

Within the limitations of the study, multiple-visit treatment was not associated with a better quality of root canal treatment compared to single-visit treatment.

## Introduction

Root canal treatment or endodontic treatment in multiple visits has been a traditionally accepted protocol [1]. The rationale for multiple-visit endodontic treatment is the use of intra-canal medication between the dental visits, which primarily aims to eliminate microorganisms and their by-products from the root canal system [2]. However, a systemic review found no significant differences between the antimicrobial efficacies reported for single-visit and multiple-visit endodontic treatment [3]. Moreover, clinical studies indicated that the success rate of single- and multiple-visit root canal treatment showed no significant difference [4-6]. The other reason for multiple-visit endodontic treatment was the amount of time required to complete the treatment [7].

With the introduction of contemporary endodontic techniques and equipments, such as magnifying devices, electronic apex locators, and engine-driven rotary nickel titanium files, the chair-side time for root canal treatment has been shortened, and the endodontic treatment can therefore be completed in a single-visit root canal treatment [8]. The concept of single-visit root canal treatment was described as early as 1880 [3]. The advantages of doing single-visit endodontics are the reduction in number of patient appointments per tooth, reduction of inter-appointment leakage, immediate use of the canal for retention of the post, particularly in the anterior region (an aesthetic consideration), reduced procedural costs, and decreased morbidity from repeated injections and rubber dam placement [9].

The primary objective of root canal treatment is to obtain success in terms of the prevention and healing of endodontic diseases [9]. The outcome of root canal treatment has been shown to be significantly associated with the technical quality of root canal fillings when judged radiographically [10]. Successful periapical healing following root canal treatment is strongly associated with adequate root canal filling [11-13]. The radiographic technical quality of root canal treatment is determined by multiple variables, such as the length of the root canal filling material in relation to the radiographic apex, the density of the root filling material (presence of voids), the taper of the canal filling, and the incidence of procedural errors [14]. Several studies have used a radiographic assessment of the technical quality of root canal treatment as a means of assessing the overall quality of root canal treatment carried out by dental students in various territories [10, 14-16].

In the past, most dental schools concentrated on teaching the multiple-visit concept. However, the procedure of single-visit endodontics is now advocated by at least 70% of dental schools [17]. To date, no study has evaluated the differences in quality of root canal treatment between single- and multiple-visit treatments done by undergraduate students. The aim of the current study was to evaluate the overall quality of single-visit and multiple-visit root canal treatment performed by undergraduate dental students at King Saud University (KSU), Riyadh, Saudi Arabia. 

## Materials and Methods

***Selection of cases***

The Ethical Committee of the College of Dentistry Research Center (CDRC), KSU, approved the design of this case-control study. The records of patients who had received dental treatment from 77 undergraduate students at the College of Dentistry, Girls University Campus (GUC), KSU, from 2012 to 2014 were collected. These records were then screened for teeth that had completed endodontic treatment. Root canal cleaning and shaping for all included teeth were carried out with the step-back technique using a stainless steel K-file (Dentsply, Tulsa, OK, USA) with 0.02 taper, and irrigation was done with 1% sodium hypochlorite solution using a syringe. Root canal filling was performed with IOS-standardized gutta-percha cones and AH-Plus sealer (Dentsply, Tulsa, OK, USA) using the cold lateral compaction technique. Teeth with incomplete documentation, missing pre- or/and postoperative periapical radiographs, or poor quality radiographs were excluded, as well as teeth indicated for the use of intra-canal medicaments. The number of visits required to complete the root canal treatment was obtained from dental records. In the undergraduate clinic, each dental visit was extended to three hours.

***Matching***

**Figure 1:**
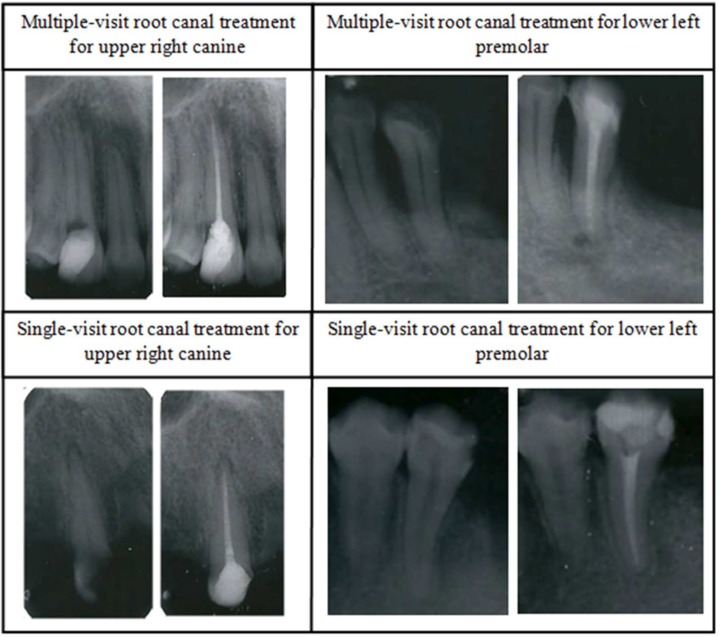
Example of matching of pre and postoperative periapical radiographs of anterior and posterior teeth treated in single and multiple-visit

Teeth were 1:1 matched so that a tooth which required single-visit treatment was matched with a tooth that required multiple-visit treatment (Figure 1). Each pair of teeth was matched by student, tooth type, and jaw quadrant. If the same tooth type or jaw quadrant was not available, the nearest match to it was selected. Furthermore, each pair of teeth was matched by the degree of case difficulty, *i.e.*, tooth curvature, presence, absence of full coverage restoration, or previous root canal filling. For example, Figure 1 shows a maxillary right canine that had minimal difficulty (no curvature or previous root canal filling) and was treated in multiple visits which is matched by another upper right canine with the same degree of difficulty that was treated by the same student in a single visit. Matching was implemented to ensure that any difference between the two groups of comparison (single-visit treatment *vs.* multiple-visit treatment) were not a result of differences in the matching variables [18, 19]. All patients were healthy and aged between 18 to 43 years old.

***Radiographic evaluation of the technical quality of root canal treatment and detection of procedural errors***

Pre- and postoperative periapical radiographs were acquired by the parallel technique using Kodak Ultra-speed D films (Care stream Health, Inc., Rochester, NY, USA). Radiographs were mounted in a cardboard slit to block ambient light from entering the illuminated viewing box (Star X-ray Illuminator; Star X-ray, Amityville, NY, USA) and examined under 2× magnification with a magnifier.

According to the endodontics case difficulty assessment form presented by American Association of Endodontists (AAE) [20], the presence of preoperative conditions (full coverage restoration, 10-30º root curvature, and previous root canal filling) was examined by means of the pre-operative radiographs. The technical quality of the root canal treatment was examined from the postoperative radiographs according to the criteria described by Barriesh-Nusair *et al. *[14], which include the obturation length, density, and taper. The length of each root canal filling was categorized as adequate (0-2 mm short of the apex), short, and overfilled based on its relationship with the radiographic apex. The density and taper of the filling were evaluated based on the presence of voids and the uniform tapering of the filling, respectively. In addition, the presence of procedural errors, such as transportation, ledge, perforation and separated instrument, was examined. Radiographic evaluation was based on the root which had the poorest canal filling quality.

***Intra-examiner reliability***

Radiographic evaluation was performed independently by two endodontists blinded to the number of treatment visits. The examiners’ evaluation scores were compared with those of a set of 15 periapical radiographs. The time that elapsed between the first and second readings was two weeks. In case of a disagreement, the two observers came to a consensus.

***Statistical analysis***

The statistical analysis was performed using SAS 9.3 software (SAS Institute Inc., Cary, NC, USA). There were five binary evaluation criteria: obturation length, density, taper, presence of procedural errors, and overall quality (acceptable quality of filling was defined as adequate obturation length, density, taper, and absence of procedural errors). To take into account the matched-pairs design, exact conditional logistic regression [21] was proposed to analyze the small set of binary data. The exact conditional score tests were used to determine if an effect was statistically significant. A *P*-value less than 0.05 indicated that an effect was statistically significant at the 0.05 level of significance. The odds ratio was estimated for the independent variables, and the corresponding 95% confidence intervals (CIs) were also computed. Kappa coefficients [22] were used to determine the intra-rater reliability of the data.

## Results

From 2012 to 2014, 973 root canal treatments of maxillary and mandibular teeth were done by undergraduate dental students. Of these, 100 (10.28%) teeth were treated using a single-visit protocol; however, 23 (2.36%) teeth were excluded from analysis 

due to un-equivalent matching criteria, missing radiographs, or lack of homogeneity of the selected tooth type. The total number of teeth evaluated in this study was 154; 77 were treated using a single-visit protocol, and 77 were treated using a multiple-visit protocol.

The kappa values for intra-examiner reliability were 1.00, 0.63, 1.00, and 0.86 for obturation length, density, taper and presence of procedural errors, respectively. 

Two-way frequency tables were used to compare the percentage of root canal treatments done in single *versus* multiple visits, in terms of obturation length, density, taper, presence of procedural errors, and overall quality (Table 1). The result of the exact conditional score tests indicated that there were no statistically significant differences between the two groups with respect to the preoperative conditions, obturation length, density, taper, presence of procedural errors, , and overall quality based on a 0.05 level of significance (Table 2). The odds ratio and the 95% confidence limits of the odds ratios for the evaluation criteria of root canal treatment are presented in Table 2. Note, if a 95% CI of the odds ratio contains 1, this suggests that the odds ratio does not have a statistically significant difference from 1 at the 0.05 level of significance. 

**Table 1:** Two-way frequency tables of number of visit and the outcome parameters of interest, obturation length, density, taper, procedural errors and overall quality. Numbers in parentheses are percentages

**Parameter**	**Criteria**	**Number of visits**	
		**Single-visit**	**Multiple-visit**
**Obturation Length**	Adequate (0-2 mm short of the apex)	74 (96.10)	70 (90.91)
	Inadequate(short>2 mm or overfilled)	3 (3.90)	7 (9.09)
**Obturation Density**	Adequate (0-2 voids)	73 (94.81)	70 (90.91)
	Inadequate (>2 voids)	4 (5.19)	7 (9.09)
**Procedural errors**	No	71 (92-21)	64 (83.12)
	Yes	6 (7.79)	13 (16.88)
**Obturation Taper**	Adequate	72 (93.51)	74 (96.10)
	Inadequate	5 (6.49)	3 (3.90)
**Overall quality **	Acceptable with no procedural errors	70 (90.91)	63 (81.82)
	Acceptable with procedural errors/not acceptable and require retreatment	7 (9.09)	14 (18.18)

**Table 2:** Exact conditional score tests of the effect of Number of visit (single and multiple visit) and presence of preoperative condition on the quality of root canal treatment

**Dependent variable**	**Independent variable**	**Exact conditional score tests**	
		***P*** **-value**	**OR (95% CI)**
**Obturation length**	visit	0.2625	2.281 (0.573, 16.745)
	Preoperative condition	0.6389	2.667 (0.250, 105.612)
**Obturation density**	visit	0.6250	0.333 (0.006, 4.151)
	Preoperative condition	1.0000	1.414 (0.013, 156.498)
**Procedural errors**	visit	0.1542	0.384 (0.051, 1.590)
	Preoperative condition	0.0604	0.153 (0.003, 1.354)
^*^ **Obturation taper**	visit	1.0000	1.000 (0.072, 13.796)
**Overall quality**	visit	0.1739	2.476 (0.569, 13.847)
	Preoperative condition	0.0832	7.221 (0.746, 380.315)

*OR=Odd ratio, CI=Confidence interval; *

* *In obturation taper, note that preoperative condition was not included in the model as including preoperative condition in the model resulted in the non-existence of the exact conditional maximum likelihood estimate*

## Discussion

A postoperative radiograph is one of the key elements assessed in determining the success of root canal therapy. Such radiographs are routinely taken as a means of assessing the quality of obturation, which has a direct bearing on the long-term success of an endodontically treated tooth [10]. The rationale of this retrospective study was to compare root canal treatment quality between single-and multiple-visit root canal treatments in undergraduate students at KSU. No statistically difference in root canal treatment quality (obturation length, density, taper, presence of procedural errors, and overall quality) was observed between single- and multiple-visit treatments.

The effectiveness of endodontic treatment regarding the treatment session is still debatable. Dentists determine the best treatment approach (single-visit *versus* multiple-visit) by considering the short-term outcomes (pain and/or swelling) and long-term outcomes (healing and success rates) after endodontic therapy [23]. A recent systemic review concluded that the success rates of single-visit and multiple-visit root canal treatments were similar, regardless of the precondition of the pulp and periapex [1]. Moreover, several studies failed to demonstrate a significant difference in the incidence of postoperative pain/flare-up between the two treatment approaches [24-26].

The presence of procedural errors (transportation, ledge, perforation, and instrument separation) causes inadequate instrumentation and/or obturation of the root canal system [27]. The incidence (7.8%) of procedural errors in teeth treated in a single visit was not significantly different from that of teeth treated in multiple visits (16.9%). This was consistent with the findings of a previous study which reported that single-visit endodontic treatment decreased the incidence of mishaps to 7%, whereas the incidence of mishaps increased to 16.2% and 28.3% for teeth treated in two and three visits, respectively [28].

The existence of preoperative conditions, such as root curvature, full coverage restoration, and previous root canal treatment, was found to reduce the quality of root canal treatment [16]. Therefore, the effect of the preoperative condition on the quality of root canal treatment was assessed in this study to ensure that any differences between the two treatment approaches were not a result of the presence of preoperative conditions (full coverage restoration, 10-30^o^ root curvature and previous root canal filling). In contrast to an earlier report [16], the results of the present study showed that there was no statistically significant relationship between preoperative conditions and the quality of the root canal treatment. This contradiction in results could be due to the small sample size of the current study and the different study designs.

The evaluation of the technical quality of root canal treatment is usually performed by experienced endodontists [29, 30]. In the present study, two experienced endodontists were asked to evaluate the quality of the root canal treatment. Kappa values of 1.00, 0.63, 1.00, and 0.86 in obturation length, density, taper, and presence of procedural errors, respectively, indicate moderate to excellent agreement among the examiners. In fact, differences in specialty training and experience strongly influence endodontic agreement and decision-making [31].

The main difference between single-and multiple-visit endodontic treatments is the use of intra-canal medicament between the visits. Nevertheless, the antimicrobial effect of intra-canal medicament is controversial [32, 33]. Calcium hydroxide is the most frequently used endodontic intra-canal medicament [34, 35]. However, the presence of calcium hydroxide residues in the canal could adversely affect the working length determination and compromise the sealing ability and penetration of the filling material in the lateral canals [36, 37]. Therefore, the teeth dressed with intra-canal medication were excluded from this study to rule out the effect of the dressing material on the quality of the root canal treatment.

The duration of the dental visit was 3 h in the undergraduate endodontic clinic; this was considered adequate for the completion of endodontic treatment in a single visit [8]. However, only 10% of the cases were completed in a single visit. This could be due to the lack of preclinical training using the single-visit root canal treatment and the use of a stainless steel hand file for canal instrumentation. The introduction of rotary nickel-titanium files and other advanced equipment in endodontics, such as electronic apex locators and magnifying devices, have shortened the time required for root canal treatment [8]. Therefore, including these advanced endodontic technologies in the curriculum of undergraduate courses may encourage the practice of single-visit root canal treatment and increase its efficacy.

The null hypothesis that there is no difference in the quality of root canal treatment between single- and multiple-visit treatments is supported by the results of this study. This lack of difference suggests that the single-visit approach of endodontic treatment is an alternative to the conventional multiple-visit treatment. Therefore, it is advocated to include the approach of single-visit endodontic treatment in the preclinical and clinical curriculum of undergraduate endodontic courses. 

## Conclusion

Within the study limitations, the single- and multiple-visit endodontic therapy performed by undergraduate students using stainless steel files showed similar technical quality of root canal treatment. 
